# Deep Belief Networks Learn Context Dependent Behavior

**DOI:** 10.1371/journal.pone.0093250

**Published:** 2014-03-26

**Authors:** Florian Raudies, Eric A. Zilli, Michael E. Hasselmo

**Affiliations:** 1 Center for Computational Neuroscience and Neural Technology, Boston University, Boston, Massachusetts, United States of America; 2 Center of Excellence for Learning in Education, Science, and Technology, Boston University, Boston, Massachusetts, United States of America; 3 Facebook, Menlo Park, California, United States of America; 4 Department of Psychology and Graduate Program for Neuroscience, Boston University, Boston, Massachusetts, United States of America; The University of Plymouth, United Kingdom

## Abstract

With the goal of understanding behavioral mechanisms of generalization, we analyzed the ability of neural networks to generalize across context. We modeled a behavioral task where the correct responses to a set of specific sensory stimuli varied systematically across different contexts. The correct response depended on the stimulus (A,B,C,D) and context quadrant (1,2,3,4). The possible 16 stimulus-context combinations were associated with one of two responses (X,Y), one of which was correct for half of the combinations. The correct responses varied symmetrically across contexts. This allowed responses to previously unseen stimuli (probe stimuli) to be generalized from stimuli that had been presented previously. By testing the simulation on two or more stimuli that the network had never seen in a particular context, we could test whether the correct response on the novel stimuli could be generated based on knowledge of the correct responses in other contexts. We tested this generalization capability with a Deep Belief Network (DBN), Multi-Layer Perceptron (MLP) network, and the combination of a DBN with a linear perceptron (LP). Overall, the combination of the DBN and LP had the highest success rate for generalization.

## Introduction

A hallmark of intelligent behavior is the controlled and flexible reuse of experience. A number of studies suggest the mammalian prefrontal cortex guides behavior based on rules generalized from experience [1], [2], [3], [4], [5], [6], [7]. The neural activity in the prefrontal cortex shows changes that depend upon sensory context and these changes in activity can be used to guide decision-making [8], [9], [10], [11]. Models of prefrontal cortex have attempted to simulate how neural circuits could provide the rules for action selection during behavioral tasks based on the context of the decision in addition to specific sensory input cues [12], [13], [14]. However, many previous models of prefrontal cortex activations and behavior focus on responses to familiar stimuli and not context-dependent responses for novel stimuli. Here, we simulate behavior for novel stimuli where correct responses can only be inferred from context. We establish this context by using two symmetries.

Generalization versus specialization is a major problem in computational learning. This is the case for supervised, unsupervised, and semi-supervised or reinforcement learning [15], [16]. Generalization can happen by using context, e.g. in the form of symmetries. Such symmetries can be represented in a structure preserving map [17], [18], [19], or symmetries might be built up in a decision tree [20], [21], [22]. We decided to capture symmetries using more biologically plausible networks, which do well in a number of tasks [23].

For the generalization through context we study three networks: A Deep Belief Network (DBN), a Multi-Layer Perceptron (MLP) network, and the combination of a DBN with a linear perceptron (LP). We explore the parameter space of these networks and chose parameters (such as the number of repetitions, number of layers, or number of hidden neurons) of well-performing networks while training with a subset of all stimuli but testing all stimuli. For networks with these fixed parameters we then further increase the subset excluded from training and again evaluate the performance by testing all stimuli. The combination of DBN and LP shows the best performance. We conclude that DBNs provide representations that allow a linear separation of outcomes including novel stimuli by extracting symmetries from presented stimuli.

## Methods

We ran all simulations in Matlab 7.12.0.635 (R2011a). The supplementary material includes scripts to replicate our figures and simulations (File S1).

### Task

**Figure 1:**
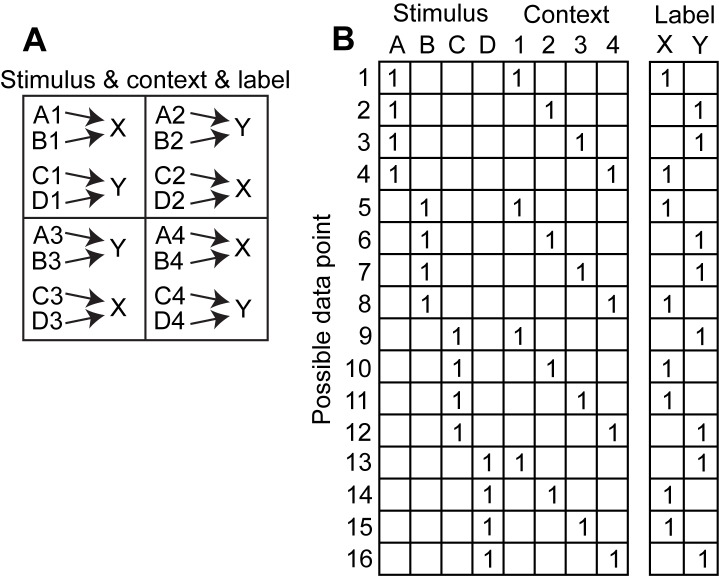
Shows the double-context task which has four stimuli A, B, C, and D in one of four contexts 1, 2, 3, or 4. (**A**) Mapping between stimuli and quadrant context onto responses X or Y, which gives 16 stimulus-context combinations or data points. (**B**) Matrix with binary, training data where stimuli and quadrant context are concatenated into one input vector with eight dimensions and X and Y into an output vector with two dimensions.

The task requires the association of sixteen stimulus-context combinations with one of two responses (Fig. 1A). Stimuli are referred to by the letters A, B, C, and D and context is referred to by the numbers 1, 2, 3, and 4. In this task responses vary symmetrically across contexts, which allows for the inference of responses to novel stimuli based on previously presented stimuli. For instance, the symmetry of response X within the 1^st^ and 4^th^ quadrant is expressed in the associations A1→X, B1→X and A4→X, B4→X. We use ‘→’ to express ‘is associated with’. If now, A1→X is a novel stimulus but all other three included in the symmetry have been presented before (along with the complementary ones C1→Y, D1→Y, C4→Y, D4→Y) then A1→X can be inferred using the symmetry. In total the task has symmetries for the 1^st^ and 4^th^ and the 2^nd^ and 3^rd^ quadrant, which we refer to as the 1^st^ context, and within the quadrant for stimuli grouped together with the same response, which we refer to as the 2^nd^ context (Fig. 1A). Because of these two contexts we call this a double-context task. To train the networks we use the four stimuli and four contexts quadrants as binary input nodes. An input node has a ‘1’ if the stimulus in a certain context is present. Otherwise the input node receives a ‘0’. We use the same binary format for the two output nodes, which we label X and Y according to the associated responses. We also refer to these labeled responses as class labels X and Y. This gives sixteen possible combinations or data points (Fig. 1B).

### Simulations

In simulations we trained the networks with 14, 13, or 12 out of 16 possible data points (the term “data points” refers to the 16 stimulus-context-response combinations used in the task). We ran tests with all 16 possible data points. Our fixed parameter set included 200 replicates of the 14 data points, which were randomly shuffled for each of the 50 epochs. These 200×14 = 2,800 data points (including replicas) were divided into batches of 100 data points. During one epoch all 28 batches were presented. Other fixed parameters used in most simulations include the use of 40 hidden neurons per layer and three layers for the DBN (except when otherwise noted). The MLP had 40 hidden neurons as well. In addition, to this fixed parameter configuration we varied one parameter at a time in the four simulations. These simulations vary the number of repetitions, vary the number of hidden neurons, vary the number of layers (not for the MLP); or they vary the data points excluded from training.

### Performance Evaluation

We evaluated the performance of the networks by reporting the error probability defined as the number of incorrect responses divided by the sum of incorrect and correct responses. In all our simulations we ran 50 repetitions with different initializations and computed the mean error probability plotted as histogram. In addition, we plotted plus/minus the standard errors using error bars superimposed onto the histogram.

### Restricted Boltzmann Machine

One neural network model used for generalization in these simulations was a deep belief network (DBN) of which each layer is a Restricted Boltzmann Machine (RBM). More generally than the RBM we assume a Random Markov Field (RMF) with the visible variables 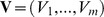 and hidden variables 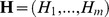. A note on nomenclature: We use non-cursive, bold face letters to indicate vectors. Uppercase letters denote random variables and lowercase letters the corresponding samples of such random variables. For the Gibbs distribution of 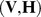 we marginalize over the hidden variables 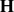 to get the marginal distribution for the observable variables 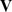:
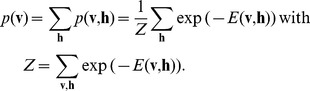
(1)


For this marginal distribution of the RMF we define the log-likelihood with hidden variables for the model parameters 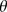:
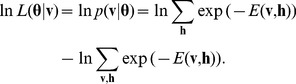
(2)


Calculating the gradient w.r.t. the model parameters 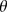 of this log-likelihood we get:
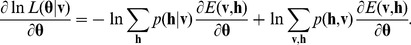
(3)


The RBM is a network consisting of two layers: A visible layer and a hidden layer, which represent the visible and hidden variables, respectively. For the RBM we assume that the network has no recurrent connections; thus, visible and hidden variables are not dependent on themselves. Visible and hidden variables 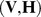 are binary, thus 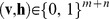. For this RBM network we chose the Gibbs distribution 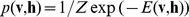 with the energy function:
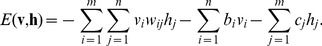
(4)


We will see that this choice of an energy function has several properties that make it possible to interpret the resulting RBM as a neural network with the weights *w_ij_* modeling the synaptic plasticity – these weights are updated only locally – and the visible and hidden variables being interpreted as neurons with a sigmoid activation function and the bias term *b_i_* and *c_j_*, respectively.

Interpreting the RBM as a graph, this graph has only connections between layers and thus visible and hidden variables are independent. Formally, we express this through:
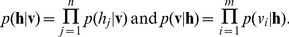
(5)


For the RBM, assuming the Gibbs distribution 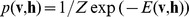 with the energy function from Eq. (4), we get the conditional probabilities:
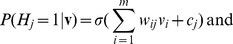
(6)

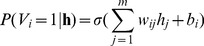
(7)with the sigmoid function 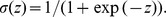 These identities can be shown using the definition of the energy function [24].

When evaluating the gradient from Eq. (3) for the energy function in Eq. (4) and using the expressions from Eq. (6) and (7) we get the following gradient updates:
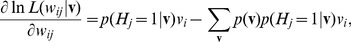
(8)

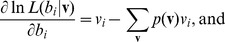
(9)

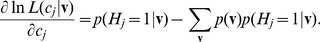
(10)


For the contrastive divergence (CD) algorithm we run a Gibbs chain for 1.5 steps starting with the sampling of 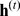 from 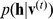. Next we use this 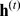 to sample 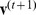 from the distribution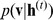 and then again 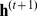 from 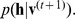 We use the endpoint of this sampled chain together with the data to update the weights and biases. For *N* data points in one batch we get the updates:
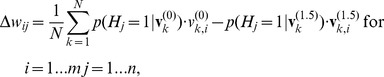
(11)

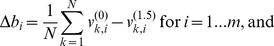
(12)

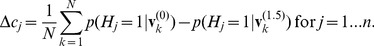
(13)


Note that we use the super-index 1.5 to denote the expansion of the Gibbs chain by 1.5 steps, essentially using a *positive phase* computing the hidden node probabilities and a *negative phase* back projecting the hidden node probabilities to the visible nodes and then computing the probabilities for the hidden nodes in another positive phase. The positive phase increases the likelihood and, therefore, is called positive while the negative phase reduces the likelihood and, therefore, is called negative. The updates from Eqs. (11) to (13) are embedded into the general learning rule for parameters 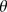 either being *w_ij_*, *b_i_*, or *c_j_*: 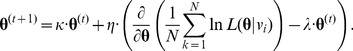
(14)


The parameter *κ* = 0.5 is the momentum term, the parameter *η* = 0.2 is the learning rate, and the parameter *λ* = 2×10^−4^ is a penalty term. For the update of these bias terms in Eq. (12) and (13) we set the penalty term *λ* = 0. We used M = 50 epochs of training. For the last M_avg_ = 5 epochs we averaged the weights and biases for the updates. If a teacher signal is present in the form of correct responses or class labels we use the same update rules from Eqs. (11) to (13) for the hidden layer.

To predict a class label for the given input data 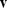 we use the log likelihood from Eq. (2) with the energy definition from Eq. (4) for each class label *j* separately:
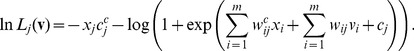
(15)


The identifier 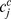 denotes the bias and 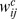 the weights linking the output of the hidden layer to the class labels. The binary variable 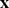 represents the likelihood of a class, e.g. when probing for the j^th^ class the j^th^ component in 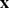 is set to one and all other components are set to zero. We select the class with the minimum log likelihood as output (winner takes all).

### Deep Belief Network

A deep belief network (DBN) can be constructed as a stack of RBMs where the hidden layer of the (i-1)^th^ RBM in the stack is the input to the visible layer of the i^th^ RBM in the stack. Training of these RBMs happens sequentially starting with the 1^st^ RBM in the stack. When the training has finished (all epochs and batches) for the first RBM in the stack an abstract output representation, also called features, of the input has formed at the hidden layer. These features are passed on to the 2^nd^ RBM in the stack and then this RBM is trained. This proceeds until the last RBM in the stack has been trained. In addition to the data from the output layer of the prior RBM the training at this last RBM happens with the correct response labels using the same rules as in Eqs. (11) to (13). Note that the information of these correct response labels is only present during the training of the last RBM. None of the other RBMs in the stack are affected by the correct response labels. Thus, training within each RBM uses forward and feedback signaling (known as *positive phase* and *negative phase*) but no feedback signaling happens across RBMs. This is different from a multi-layer perceptron whose weights are adapted through back propagation. DBNs found many applications [23], [25].

### Multi-Layer Perceptron Network

Another network that we used to test generalization was the multi-layer perceptron (MLP). The MLP network undergoes adaptation of its weights using a gradient descend approach like the RBM. A two layer perceptron network is powerful enough to approximate functions or solve classification tasks that are not linear separable [26]. Thus, we chose a two-layer network: The layer between input and hidden nodes and the layer between hidden and output nodes. The *m* input nodes receive the input 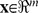. These inputs are connected through the weights 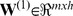 to the *h* hidden nodes, which have the bias 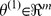. These hidden nodes have a nonlinear transfer function *f*. Thus, the signal flow through the first layer from the input to the output of the hidden nodes is:
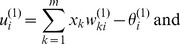
(16)

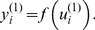
(17)


Similarly, the signal is transferred from the hidden to the output nodes through the weights 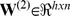 and passed through a nonlinear transfer function *f* in the output nodes, which have the bias 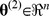. The signal flow from the outputs of the hidden nodes to the outputs of the output nodes is:
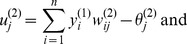
(18)

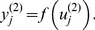
(19)


The weights of the two layers are adapted using a least square optimization for the output error assuming the teacher signal 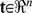:
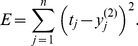
(20)


Calculating the gradient of the energy functional *E* with respect to the variables 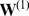, 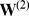, 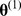 and 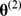 and using a least gradient descend approach when updating these variables we get 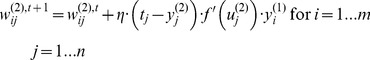
(21)

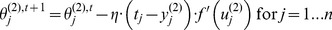
(22)

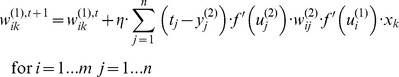
(23)

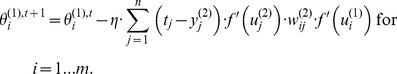
(24)


We absorbed factors of two into the learning rate *η* = 0.1 and used the sigmoid function 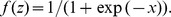 For training we used M = 50 epochs each consisting of 200 repetitions of the original data in randomly shuffled order. For 14 data points we have 14×200 = ,800 data in one epoch, which were presented sequentially. So we do not use batch learning. Instead the weights and thresholds are updated using Eqs. (21) to (24) after the presentation of each data point.

### Linear Perceptron

The Linear Perceptron (LP) is a single node “network” receiving the inputs 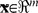, has the weights 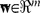, and the threshold 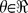. This LP has two outputs: ‘1’, which we associate with the response X, and ‘0’, which we associate with the response Y. For ease of the formulation of the learning rule we included the threshold in the weights by adding it as a last component. Accordingly, the input vector also has an added component, thus 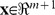 and 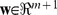. Using this formulation, the output of the LP is:
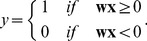
(25)


Assuming that outputs and labels (responses) assume values 0 or 1, the learning rule for the weights and threshold is 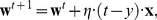
(26)with the learning rate *η* = 0.01, the teacher signal *t*. We run this learning rule at most for 1,000 iterations.

The LP is trained based on the representation of the last RBM in the DBN. The output activation of the DBN's last layer is weighted using the weights for the class labels and summed together. This weighted sum together with the label information is forwarded to the LP. The LP is trained after the DBN, in sequence, and only with the stimulus-context combinations that were also used to train the DBN.

## Results

We divide the results section into three parts. The first part explains the internal representations created in different layers of the DBN and its interface with the LP. The second part presents the results of our parameter tuning procedure on performance of each of the three types of networks: 1. DBN, 2. MLP and 3. DBN and LP. The third part compares the training of the three types of networks DBN, MLP, and DBN and LP with 14 or fewer out of the 16 data points to study generalization using context information implicitly contained in our task.

### Representations

**Figure 2:**
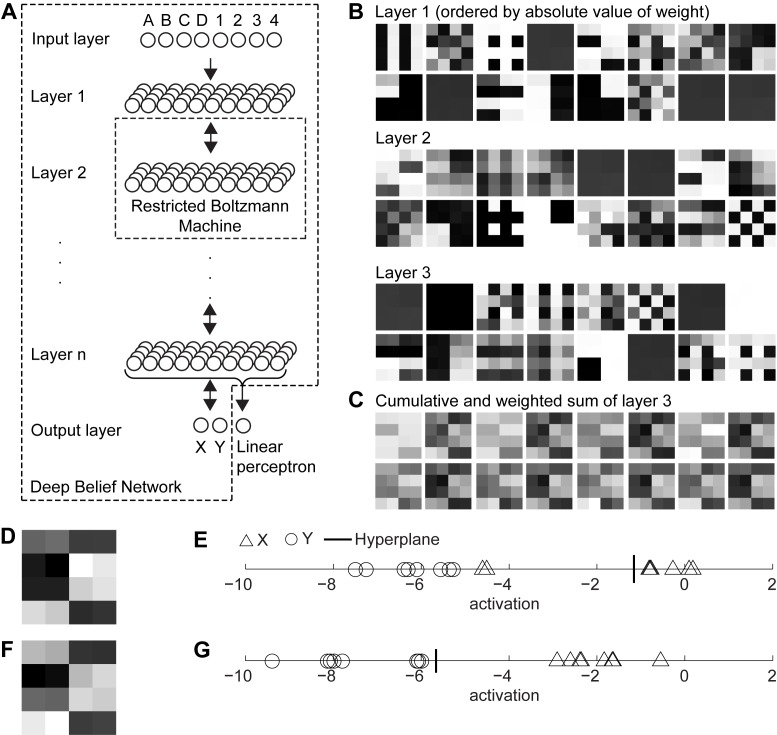
Shows the network architecture for the Deep Belief Network (DBN) combined with the Linear Perceptron (LP) together with the internal representations of these networks. (A) Network architecture of an N-layer DBN. (B) Internal representation for a 3-layer DBN when probing with the 16 stimulus-context combinations or data points. (C) Cumulative and weighted sum of the 16 strongest weights for the output node X of the DBN. (D) A rescaled version of the 16^th^ tile, which shows that all data points that map to class X have high values (brighter) and those data points that map to class Y have low values (darker). (E) Inputs to the LP. The threshold for classification is denoted as bold, vertical line when trained without A1 and B1 and (G) when trained with all data.

The DBN extracts features and “meta”-features of the original data within the layers of its network. For our task we chose the separate input nodes for the stimuli A, B, C, and D and for the quadrant contexts 1, 2, 3, and 4 (Fig. 2A). In our “standard” configuration these inputs are forwarded to the first layer with 40 hidden variables. Only the n^th^ layer is connected to the outputs X and Y. When the LP is present in the network this n^th^ layer connects also to the LP (Fig. 2A–curled bracket). In the DBN, the first layer extracts features from the inputs (Fig. 2B–layer 1). The second layer extracts features of these features (Fig. 2B–layer 2) and the third layer extracts again features of features (Fig. 2B–layer 3). Taking the cumulative sum of the strongest components weighted by the output weights for X of the last layer in the DBN shows a representation according to the input stimulus and context, here 16 combinations (Fig. 2C). In this representation all combinations of stimulus and context for X have a high value (ligher shades) and those for Y have a low value (darker shades in Fig. 2D and 2F). These values of the weighted sum together with their class labels (response) X or Y are then used as the input for the LP. When trained with 14 out of 16 data points, with A1 and B1 excluded, the LP classifies these two A1 and B1 wrongly as Y instead of X for the initialization examples shown (Fig. 2E). When training and testing with all data points all input combinations are correctly classified (Fig. 2G).

### Parameter Tuning

**Figure 3:**
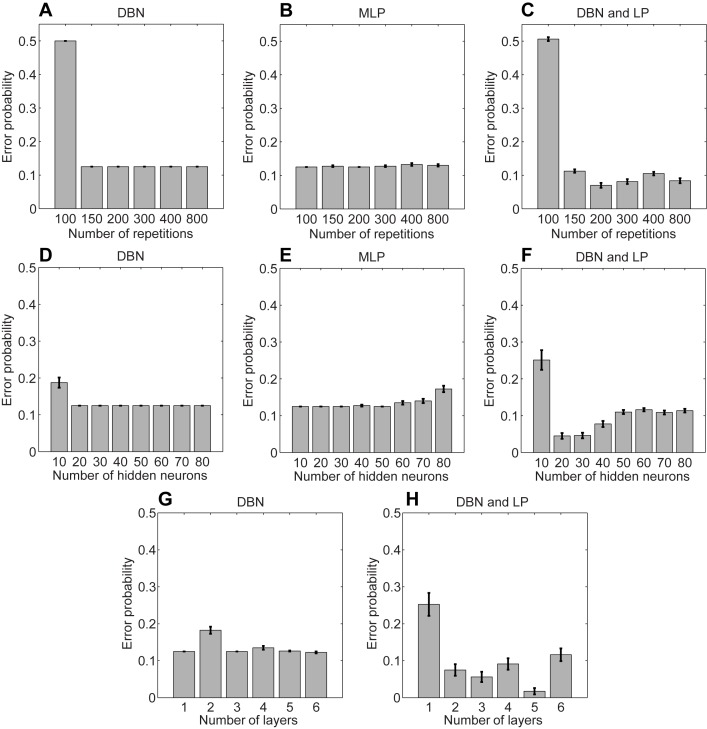
Simulation results for parameter tuning. In all simulations the default parameters if not varied are 200 repetitions, 40 hidden neurons, and 3 layers. We report the histogram (mean) and bars (standard error) for the error probability using 50 runs. (A–C) Error probability for the Deep Belief Network (DBN), Multi-Layer Perceptron (MLP), and DBN combined with a Linear Perceptron (LP) when varying the number of repetitions, (D–E) or when varying the number of hidden neurons, or (G and H) when varying the number of layers. In the last case only the DBN and DBN with LP are included because we used always two layers for the MLP.

We studied the performance of the three types of networks when varying parameters of the networks. The free parameters that we varied were the number of repetitions of the input data points within each epoch, the number of hidden neurons, and the number of layers when the DBN was involved. As a measure of evaluation we use the mean error probability for 50 runs. All simulations show the performance after we trained the networks without A1 and B1 and tested with all data points. For the DBN and MLP everything more than 100 number of repetitions provides an error probability of 0.125 = 2/16, essentially classifying the response to A1 and B1 in an incorrect manner (Fig. 3A and 3B). Adding the LP to the DBN reduces the error probability further below this probability of 0.125 (Fig. 3C). For some initializations of the DBN the LP finds a threshold that separates both classes X and Y correctly.

A number larger than 10 hidden neurons for the DBN reduces the error probability to 0.125 (Fig. 3D). Using a number larger than 50 hidden neurons in the case of the MLP leads to overtraining, where the network starts to learn the presentation sequence rather than the data points (Fig. 3E). For the combination of DBN and LP a number of 20 to 30 hidden neurons appears optimal (Fig. 3F).

When the DBN is involved we vary the number of layers from one to six. For only the DBN the error probability is around 0.125 in most cases (except for two layers) (Fig. 3G). When adding the LP to the DBN network we achieve better results when using more than one layer in the DBN (Fig. 3H). Essentially, more layers provide a linear separable representation for the responses (class labels).

### Generalization through Context

**Figure 4:**
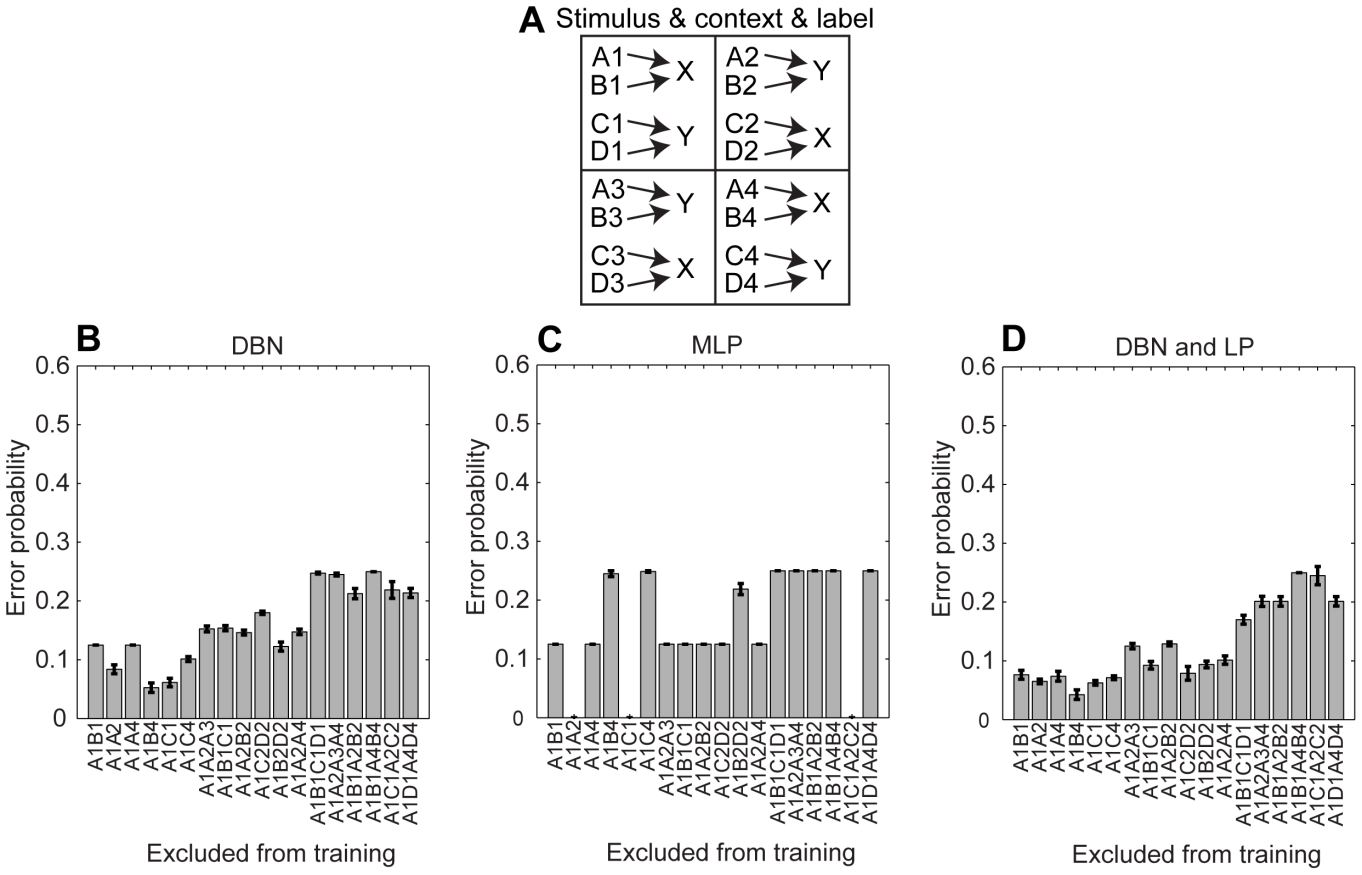
Shows the generalization capabilities of networks. (A) A depiction of our behavioral task. (B–C) Error probability for the Deep Belief Network (DBN), Multi-Layer Perceptron (MLP) network, and DBN with Linear Perceptron (LP), respectively.

Through symmetries of the context provided within each quadrant and across quadrants, all combinations could be learned even when only training with a subset of all data. That is, the network could generalize correct responses to previously untrained stimuli based on the symmetries of the trained stimuli. In this series of simulations we systematically exclude two, three, or four data points from the training set and evaluate the error probability. As expected the error probability increases when removing more data points from the training set (Fig. 4B, 4C, and 4D). For the MLP, removal of some combinations of stimulus and context can be learned, e.g. A1, A2; A1, C1; or A1, C1, A2, and C2 (Fig. 4C) – because of the default response of the MLP being correct. Adding the LP to the DBN helps to improve the error probability (compare Fig. 4B with 4D). This demonstrates that the symmetries in the training set allowed generalization to previously unseen combinations of stimuli and context that could be used as probe stimuli in a behavioral task.

## Discussion

We modeled the double-context task, a highly structured behavioral task using a set of 16 stimulus-context-response combinations. While the DBN and MLP could learn the task without error when all data points (stimulus-context-response combinations) were used for training, the DBN and MLP failed to learn all data correctly when two data points were left out from training. Adding an LP to the DBN reduced the error probability when training with a subset of all data points, e.g. leaving out two, three, or four data points. The DBN provides a generalized representation for the task, which in certain cases can be used by the LP to correctly classify all input data points as a linear separable problem.

We could have hand-crafted features to learn the task directly with an LP, but instead we asked whether DBNs could provide such features automatically. Modified versions of reinforcement learning are alternatives to learn the double-context task [15], [17], [19], [20], [21], [22]. Here, we selected DBNs for their simplicity, biologically plausible form, and success in solving many problems [25].

Similar context tasks probed for brain areas involved in the acquisition and usage of conceptual knowledge [27], [28]. Kumaran et al. [27] use a weather forecast task: Sun or rain depends on the object and location or the presence of two specific objects regardless of their location. Four objects may each appear in one of three locations (4^3^ = 64 combinations); however, only eight of these combinations are used for training. In four of them the spatial location of an object is sufficient to predict the outcome. In another four the presence of an object pair is sufficient to predict the outcome. Probes are constructed where object pairs or object in a certain location determine the response (determined probe trails) or where object pairs with one object in a certain location indicate different responses (undetermined probe trials). Parahippocampal cortex, amygdala, posterior cingulate cortex (PCC), ventral striatum, and ventromedial prefrontal cortex (vMPFC) were correlated with the probability for success in learning the weather prediction task. The left hippocampus, vMPFC, and PCC had a positive correlation with the participant's performance during probe trials. The activation in the hippocampus, and vMPFC was significantly greater for determined probe trails than for undetermined probe trials. This data suggests that vMPFC is involved in the acquisition of conceptual knowledge. Our double-context task did not distinguish between determined and undetermined probe trails; instead the outcome was always dependent upon quadrant context and stimulus. Thus, our task only included determined probe trails. Our modeling work does not yet make any statements about the specific brain areas that are involved nor does it use any anatomical or physiological information about specific brain areas. From preliminary simulations performed with the MLP and DBN presented here, we found the networks were able to learn the weather prediction task.

We also tested a variant of our double context task for incremental training. In this variant we added a set of four more stimuli E, F, G, and H. Each of these stimuli can appear in one of four quadrant contexts 1, 2, 3, or 4 forming the same rules as stimuli A, B, C, and D (see Fig. 1a). In the incremental training procedure we learned the original 16 data points and then the added 16 data points. In the non-incremental training procedure we learned all 32 data points at once. For a medium range of repetitions (>100 and ≤200) incremental training has a lower error rate than non-incremental learning, learning all responses from the 2^nd^ set while the non-incremental training predicts these responses form the 2^nd^ set at chance level. However, for a larger number of repetitions (>200) the non-incremental training has a lower error probability. In this case, the incremental training “unlearns” the 1^st^ set after learning the 2^nd^ set. Thus, incremental training is not profitable in our modified double-context task when using the DBN and LP.

Badre et al. [28] in their task ask for an abstract label 1, 2, or 3 as response, which depends on the shape, orientation, or a colored frame surrounding a presented figure. Two sets of three object shapes, which can appear in three orientations and are surrounded by one of two colored frames, are used to define the stimuli (2×3×3×2 = 36 combinations). Their study focused on the learning of hierarchical rules. In one setting the learned rule is flat; an association between shape, orientation, color, and response has to be learned. In another setting the rule is hierarchical where the color indicates if the shape or orientation information determines the response. During learning, rostro-caudal frontal brain regions were activated. Teasing apart the learning of flat and hierarchical rules shows an early activation in pre-premotor cortex. Again, our modeling work does not address specific activation patterns of specific brain regions. Our task included hierarchical knowledge in terms of the quadrant (Fig. 1A) and in terms of a rule switch, but such knowledge never determined the response directly. From preliminary simulations we know that the DBN and MLP presented here can also learn the orientation task.

For both tasks [27], [28], the authors report performance in correct responses over trials which reaches ceiling performance after a moderate number of trials (40–360). Thus, the authors of [27], [28] conclude that there is fast learning of conceptual knowledge. In contrast, our proposed networks take many more trials for learning — 2,800 instead of 40–360. Thus, for our task we advocate a slow learning process, as has been reported for memory consolidation [29], [30].

This research can be extended in a number of ways. First, our past work modeling psychological memory systems in reinforcement learning [14], [31], [32] introduced state spaces with similar sets of symmetries as in the present task, suggesting a DBN might also improve performance in those tasks. A second and more general way to extend this research is to study the learning of the location and context as well. Here we focused on the learning and generalization of the double-context task when training with incomplete stimuli. In an advanced variant of the double-context task one might also learn context and stimuli, and especially their difference. For instance, one possibility is to work with visual representations (like [27], [28]) and have the network extract and separate stimulus and context.

In summary our results show that DBNs can be a powerful tool to be combined with simpler learning techniques like the LP to provide representations that generalize using only a subset of all data points to a correct classification of all data points. This provides a potential means of understanding the neural representations that could allow generalizations based on symmetries in behavioral tasks consisting of specific combinations of stimulus and context with correct responses.

## Supporting Information

File S1 **Contains Matlab scripts to replicate our figures and simulations.**
(ZIP)
